# Agent-Based Spatiotemporal Simulation of Biomolecular Systems within the Open Source MASON Framework

**DOI:** 10.1155/2015/769471

**Published:** 2015-03-22

**Authors:** Gael Pérez-Rodríguez, Martín Pérez-Pérez, Daniel Glez-Peña, Florentino Fdez-Riverola, Nuno F. Azevedo, Anália Lourenço

**Affiliations:** ^1^Escuela Superior de Ingeniería Informática (ESEI), Edificio Politécnico, Universidad de Vigo, Campus Universitario As Lagoas s/n, 32004 Ourense, Spain; ^2^LEPABE, Department of Chemical Engineering, Faculty of Engineering, University of Porto, Rúa Dr. Roberto Frias, 4200-465 Porto, Portugal; ^3^Centre of Biological Engineering (CEB), University of Minho, Campus de Gualtar, 4710-057 Braga, Portugal

## Abstract

Agent-based modelling is being used to represent biological systems with increasing frequency and success. This paper presents the implementation of a new tool for biomolecular reaction modelling in the open source Multiagent Simulator of Neighborhoods framework. The rationale behind this new tool is the necessity to describe interactions at the molecular level to be able to grasp emergent and meaningful biological behaviour. We are particularly interested in characterising and quantifying the various effects that facilitate biocatalysis. Enzymes may display high specificity for their substrates and this information is crucial to the engineering and optimisation of bioprocesses. Simulation results demonstrate that molecule distributions, reaction rate parameters, and structural parameters can be adjusted separately in the simulation allowing a comprehensive study of individual effects in the context of realistic cell environments. While higher percentage of collisions with occurrence of reaction increases the affinity of the enzyme to the substrate, a faster reaction (i.e., turnover number) leads to a smaller number of time steps. Slower diffusion rates and molecular crowding (physical hurdles) decrease the collision rate of reactants, hence reducing the reaction rate, as expected. Also, the random distribution of molecules affects the results significantly.

## 1. Introduction

Microbial chemical factories have become an increasingly important industrial platform, with numerous applications in the food, agriculture, chemical, and pharmaceutical industries [1–5].

Recent advances in protein engineering, metabolic engineering, and synthetic biology have revolutionised our ability to discover and design new biosynthetic pathways and engineer industrially viable strains [6–8]. Metabolic engineering offers ways to enhance the yield and productivity of target compounds while combinatorial biosynthesis enables the creation of novel derivatives [9–11].

The interplay of mathematical modelling and* in silico* simulation with laboratory experiments is thus pivotal to elucidate the basic, and presumably conserved, design and engineering principles of the biological systems [12]. Understanding the behaviour of a biological system, whether it is natural or engineered, requires models that integrate the various interactions that occur at different spatial and temporal scales. However, modelling the various scales and the intra- and interscale interactions of a biological system is extremely complex and is considered an open and active area of research [13–15].

Researchers are looking into novel approaches for abstraction, for modelling bioprocesses that follow different biochemical and biophysical rules, and for combining different modules into larger models that still allow realistic simulation with the computational power available today.

This paper explores the potential application of agent-based modelling to such complex modelling. Notably, the aim is to develop a computational infrastructure for multiscale biomolecular modelling and simulation based on common biochemical and biophysical rules. The novelty of the work lays on fully considering the spatial location of the molecules and allowing for the description of intricate microscale structures, which enables the modelling of microbial behaviour in more realistic and complex environments. The prototype of the agent-based cellular simulator was developed in the open source Multiagent Simulator of Neighborhoods (MASON) [16]. The rationale behind the use of MASON, among existing agent-based frameworks, lies in its general purpose and thus the ability to support various levels of social complexity, including different physics and agent logic. Moreover, there is a project working on the development of a distributed version of MASON, which will certainly be required in order to simulate complex metabolic models and, in the future, whole cell models.

Test and validation experiments addressed the correct formulation of diffusion coefficient and reaction rate principles. Then, a simple cellular system was formulated, encompassing most of the rules previously validated and accounting for a realistic number of participants. This experiment exposes the computational requirements imposed by a realistic scenario and raises discussion about future lines of research and development for agent-based biomodelling.

The next sections of the paper describe the biological and computational rationale behind our simulator.  Section 2 summarises the key points of agent-based modelling and earlier application of agent-based models to biological systems.  Section 3 describes the biochemical and biophysical rules that guided the modelling.  Section 4 presents the agent-based model and explains the structure and functionality of the different interacting agents involved in the system. In Section 5, simulation results are compared to experimental results and noise is discussed. Final conclusions resume current achievements and draw main guidelines for future work.

## 2. Agent-Based Models and Their Application in Biology

Agent-based modelling (ABM) is as a relatively new paradigm for engineering complex and distributed intelligent systems [17]. Typically, this approach is considered suitable for scenarios where there is a population of heterogeneous individuals, which display varied and adaptive behaviour.

Generally, agents can be defined as computer systems that are situated in some environment and that are capable of autonomous action in this environment, based on mechanisms and representations somehow incorporated. The early work of Wooldridge [18] described the general characteristics of an agent as follows: autonomy, that is, agents can make decisions about what to do without direct external intervention of other systems; reactivity, that is, agents are situated in an environment, can perceive it (at least to some extent), and are able to react to changes in it; proactiveness, that is, agents do not simply react to changes in the environment but are also able to take the initiative; social ability, that is, agents can interact with other agents and participate in social activities.

In ABM, the purpose is to “monitor” the behaviour of the agent from the perspective of the agent itself, rather than the system as a whole [19]. Each agent class has multiple manifestations in the form of a population of agents that interact in the shared environment. Differing local conditions lead to different behavioural trajectories of the individual agents, and the heterogeneous behaviour of individual agents leads to the aggregated system dynamics. This process enables the generation of a population of behavioural outputs from a single model, producing system behavioural spaces consistent with population-level biological observation. Moreover, new information (e.g., finer degree of detail) can be added either through the introduction of new agent classes or by the modification of existing agent rules without having to reengineer the entire simulation.

An agent-based model (i.e., the automaton) is thus composed of agents (autonomous entities), rules (logic or mathematical), a simulation environment (source of local information), and a set of initial and boundary conditions. Agents may be defined at multiple scales, and the model can formalise the various behaviours through which individuals interact with one another, directly or indirectly, through the shared environment. This requires the preparation of plausible and adequately detailed design plans for how components at various system levels are thought to fit and function together.* In silico* results should then be validated against experimental outputs to reconcile different design plan hypotheses and render a realistic view of the system.

Such individual-based modelling has the potential to replicate cellular systems at its minimum components and thus help to understand the linkage from molecular level events to the emerging behaviour of the system [20–24]. In particular, the spatial nature of most agent-based models puts emphasis on behaviour driven by local interactions, which matches closely with the mechanisms of stimulus and response observed in biology. The Epitheliome, a representation of the growth and repair characteristics of epithelial cell populations, is probably one of the earliest applications [25]. Other more recent applications relate to biofilm formation [26], bacterial phenotypic switching [27], cancer development [28, 29], bacterial virulence in surgical site infection [30], the development of restenosis in blood vessels [31], oxygen metabolism in aerobic-anaerobic respiration [32], and the design of cellulase systems [33].

It is reasonable to say that ABM has become a popular biomodelling approach and the new models are reaching out for increasingly more complex and higher resolution problems. The key challenge is to be able to reproduce different scales realistically, in terms of the number and type of participants involved and the events taking place, whilst balancing the requirements of extendible model granularity with computational tractability. So far, the use of general purpose graphical processing unit (GP GPU) technology and multicore CPU processors are the favoured approaches to parallelise simulation algorithms [34, 35].

## 3. A Novel Agent-Based Spatiotemporal Biomolecular Model

### 3.1. Modelling Environment and Overview

A multiscale agent-based model mimicking the biology of biochemical reactions was developed using MASON version 16 [16], a Java based, open source, ABM framework that facilitates model development. This model includes agents representing common biochemical players such as metabolites, cofactors, and enzymes. The model also considers one physical barrier representing the cell membrane and a number of geographical hurdles accounting for the molecules known to exist in the intracellular space but not represented individually (to avoid unnecessary computational complexity).

The agent-based model is created on a continuous two-dimensional environment, which corresponds to 5 *μ*m^2^. The distance unit is given by the radius of the smallest molecule represented in the model and corresponds to 0.323 × 10^−3^ *μ*m. This value also establishes the upper limit for the velocity at which agents may move, such that the smaller the radius of the molecule is the faster it will move, and vice versa. The correspondence between simulation time steps and real time is configurable and may be used to validate different results.

The intracellular environment can be populated by enzymes, some metabolites, and cofactors (e.g., NAD^+^ and NADH). Once the simulation starts, other types of agents, such as other metabolites, appear in the model in accordance with the behavioural rules. So, the simulation only requires defining the particle radius and diffusion coefficient for each species and the initial number of the molecules. Agents are then distributed randomly and may circulate freely.

**Figure 1:**
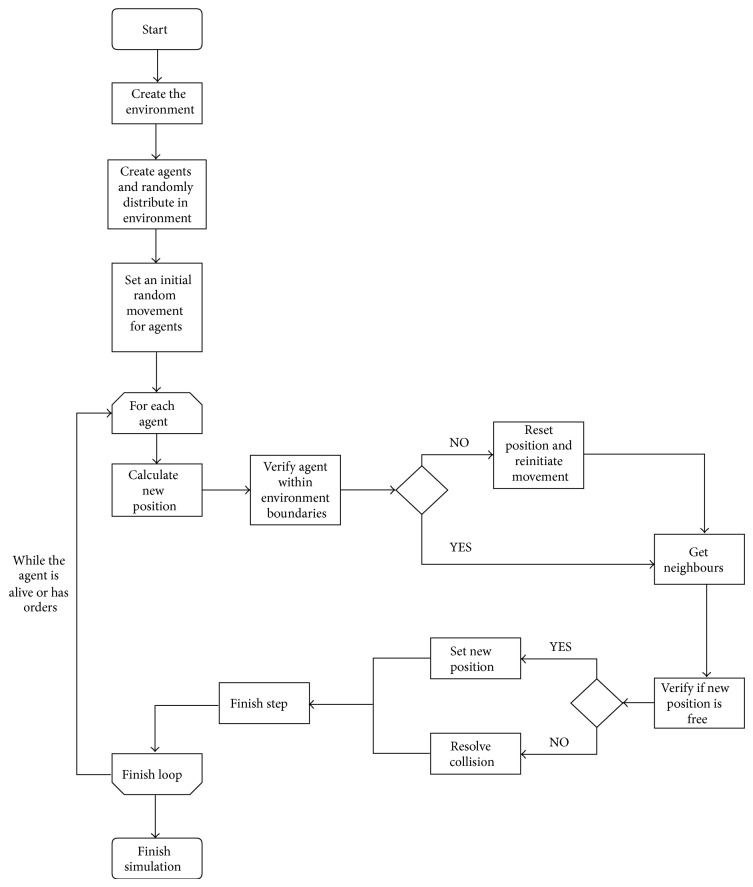
Decision process for the movement and rotation of an agent during the simulation.

Every agent, except obstacles, is randomly initialised with a given orientation. The behaviour of each agent is determined by the corresponding set of behavioural rules and most notably its spatial location (Figure 1). In each time step, the model checks the current situation of the agents and determines which rule(s) should be executed and what input values should be used.

Given the circular shape of the agents, the detection of a collision between agents is based on the Pythagorean Theorem for triangles. That is, collision is detected by knowing that if the distance between the centres of the agents is less than their combined radius the agents are to collide.

In the event of a collision, the simulator identifies the types of the agents involved and looks for any behavioural rules that may apply. Either no rule applies and agents should be reoriented or the matching rule should be executed and agents should be affected accordingly. Agents are reoriented based on the angle of collision and the corresponding diffusion rate [36, 37].

Most of the rules applicable in a scenario of collision involve enzymes and metabolites, that is, the possible occurrence of an enzymatic reaction (see Section 3.2). The reaction probability is given by the probability of the collision and the probability that a reaction occurs given that a collision is occurring. The claim of the simulation is that it can reproduce the macroscopic enzymatic rate constants *k*_*m*_ and *k*_cat_ (mass action kinetics) in homogeneous conditions.

Particularly, the number of agents representing metabolites and enzymes needs to be compared with values reported in the literature. For this purpose, at model construction, we established a conversion mechanism, between the number of agents in the simulation and the number of moles calculated in laboratorial experiments. In the literature, values of molecules are typically represented as a concentration (e.g., mM). Molar concentrations can be modified to number of molecules per volume unit by simply multiplying the concentration by the Avogadro number. Because this is a 2D simulator, a height also had to be indicated. This was assumed to be 0.005 *μ*m, an approximate typical height of an enzyme. The number of molecules in the simulator can then be obtained multiplying the previous value by the area of the simulation space and the estimated height. The diffusion rates and the sizes of the molecules can be generally obtained directly from the literature or extrapolated using some already known correlations. In our case, diffusion was calculated based on [38], which estimates molecular diffusion based on the molecular mass of the species.

Each type of agent can be tracked continuously in one run of simulation. To facilitate the inspection and a dimensional representation, distinct agent types are associated with different colours and sizes.

### 3.2. Behavioural Rules of Agents in the Model

The behavioural rules are twofold: interaction of agents with their environment and responses to the presence of other agents (Table 1). Specifically, agents interact with the cell membrane (the physical boundary of the cell) and with the obstacles in the intracellular space. The cell is able to retrieve substrates from the extracellular space and release products to this space. Other internally produced metabolites are to remain within cell boundaries.

The obstacles aim to mimic the presence of other lower level molecules in the intracellular space. They are not represented individually to preserve computational tractability (e.g., a bacterial cell contains approximately 2*E* + 10 molecules of water) but some form of representation was still required in order to model the diffusion rate and the orientation of the movement of the simulated molecules adequately.

In certain cases, the type of agent can be changed and consequently, the corresponding behavioural rules of the new type would be applied to the agent. This transition is typically based on the spatial location of the agent, its type, and the local environment. One example of agent type reassignment is the “recycling” of NADH molecules to NAD^+^ molecules whenever NADH agents collide with the cell membrane.

Regarding agent interaction, enzymes interact with cofactors and metabolites. Many enzymes require the assistance of cofactors in biochemical transformations. When an enzyme agent and a cofactor agent collide, the enzyme checks whether it requires the cofactor to operate. If so, a new agent representing the enzyme-cofactor complex (holoenzyme) is created in replacement of the two agents.

The interaction between the enzymes (or enzyme-cofactor complexes) and metabolites represents catalysis and was modelled according to Michaelis-Menten equation (see kinetic parameters section). In general, metabolites and enzymes are supposed to react within a certain probability whenever they collide, and the enzymatic reaction may be concluded in the same time step or after a number of time steps.

After the enzymatic reaction takes place, that is, the enzyme-cofactor complex collides with a substrate, the complex is destroyed and the agents representing the enzyme and the cofactor are created again. Likewise, the agents representing the substrate disappear and new agents are created for the products of the reaction.

### 3.3. Kinetic Parameters

The critical part of developing our model was related with incorporating kinetic information on the cellular dynamics, especially on different enzymatic reaction kinetics.

Generally, the kinetic scheme representing an enzymatic reaction under steady-state conditions is written as

(1) $$\begin{array}{c} \text{E}+\text{S}\overset{K1}{\underset{K-1}{⇄}}\text{ES}\overset{K2}{\underset{K-2}{⇄}}\text{E}+\text{P}, \end{array}$$

where E represents the enzyme, S is the substrate, ES is the enzyme-substrate complex, and P is the product. The association rates *k*1 and *k*2 and the dissociation rates *k* − 1 and *k* − 2 account for the substrate binding and product release forward and reverse processes, respectively.

Since the rate constants for the binding and unbinding reactions are either often unknown or difficult to determine, modelling has to rely on approximations, also called aggregate rate laws, such as the Michaelis-Menten kinetics [39, 40]:

(2) $$\begin{array}{c} V=\frac{V_{\max}\left[\text{S}\right]}{K_{m}+\left[\text{S}\right]} \end{array}$$

or, following the Lineweaver and Burk linear transformation,

(3) $$\begin{array}{c} \frac{1}{V}=\frac{K_{m}}{V_{\max}}\times \frac{1}{\left[\text{S}\right]}+\frac{1}{V_{\max}} \end{array}$$

which represents the reaction rate at a substrate concentration [S]. *V*_max⁡_ is the maximum rate that can be observed in the reaction, considering the substrate is in excess. The Michaelis constant *K*_*m*_ is a measure of the concentration of substrate at which the rate of the reaction is one half its maximum, *V*_max⁡_. That is, *K*_*m*_ is a relative measure of the affinity of the enzyme for the substrate (how well it binds). Small *K*_*m*_ means tight binding and large *K*_*m*_ means weak binding.

The turnover number

(4) $$\begin{array}{c} k_{\text{cat}}=\frac{V_{\max}}{\left[\text{E}\right]} \end{array}$$

where [E] equals total enzyme concentration, represents the number of moles of product produced per number of moles of enzyme per unit time, and is expressed in units of inverse time (*s* ^−1^). That is, the rate of the reaction when the enzyme is saturated with substrate.

Having in mind the biological meaning of the parameters, we hypothesised that the percentage of reactive collisions between enzyme and metabolite and the number of simulation time steps could together be used to mimic the rates expressed by *k*_*m*_ and *k*_cat_. To corroborate this hypothesis we conducted a series of experiments trying out different percentages of collision with reaction and number of time steps and calculating the theoretical values of *k*_*m*_ and *k*_cat_. The combination of simulation parameters was further validated against experimental values retrieved from the enzyme database BRENDA [41].

## 4. Results and Discussion

To actually demonstrate that our design plan is functionally plausible, we recreated different scenarios of enzymatic activity to show that the constructed model exhibits behaviours that match those observed in the laboratory experiments.

We present results obtained for the simulation of a simple scenario where an enzyme catalyses one substrate and releases one product. These results are discussed theoretically in terms of enzyme affinity to substrate and catalytic efficiency and further tested against experimentally calculated kinetic parameters.

Then, we show that the tool is able to model biochemical pathways, accounting for biochemical and biophysical laws adequately. We describe the computational costs of representing more complex biomolecular scenarios. We discuss a number of present commitments and simplifications necessary to ensure computational tractability and point out ongoing lines of work.

### 4.1. Approximation of Kinetic Parameters

This process of analysis is somewhat similar to that performed in laboratory experiments. That is, we studied the behaviour of an identical amount of enzyme in the presence of increasing concentrations of substrate and measured the velocity of reaction by determining the rate of product formation. Furthermore, we tested different (combinations of) simulation parameters, namely, the percentage of collisions producing reaction and the number of time steps taken by a reaction. Based on the interpretation of the Lineweaver-Burke plot, which describes the Michaelis-Menten laws for kinetic dynamics, we calculated the theoretical values of *k*_*m*_ and *k*_cat_.

Substrate concentrations ranged from 6.64*E* − 02 mM to 6.64*E* − 01 mM (200 molecules to 2000 molecules, approximately) and there is a concentration of enzyme of 4.98*E* − 02 mM (150 enzymes, approximately). Simulation parameterisation mimicked “extreme” scenarios (i.e., 100% immediate reactive collisions, very slow reactions, and barely any reactions occurring) as well as more common scenarios (i.e., midrange percentages of reactive collision and reaction duration). Moreover, the experiment was replicated 3 times (3 simulation runs for every concentration of substrate) and results were averaged.

The model assumes that a simulation tick corresponds to a configurable, specific amount of time in the system. Notably, our approach to real time-time step conversion focused on framing realistic values of velocity of reaction and hence of *k*_cat_. Regardless of the range of values of *k*_cat_ to be simulated, we should be able to represent such enzyme dynamics accurately by adjusting the equivalence in time steps.

**Figure 2:**
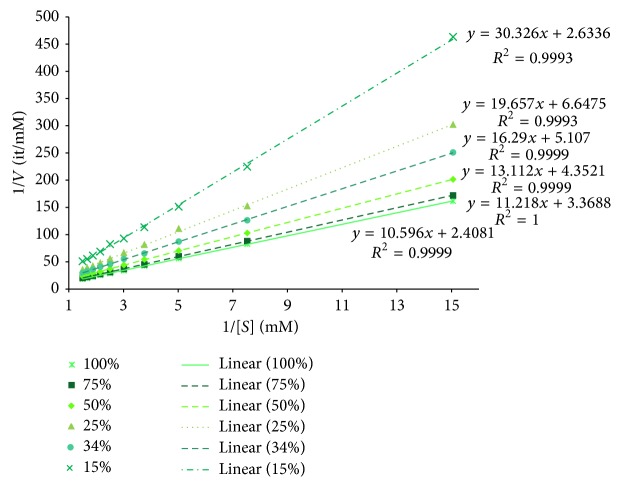
The Lineweaver-Burk plots obtained while simulating different percentages of reactive collision and a time step of 1. The markers represent the outputs of simulation and the lines represent the corresponding trend lines based on linear regression (equation also shown).

Figure 2 shows the Lineweaver-Burke plots resulting from experiments considering the duration of the reaction constant and equal to 1 time step representing 10 seconds. Since the number of time steps that a reaction takes to be concluded is considered somewhat equivalent to the velocity of the reaction, the Lineweaver-Burke plots should converge to a similar *y*-intersect. Likewise, different percentages of reactive collision should describe different affinities of the enzyme for the substrate, and this effect should be reflected in the slope of the plots. Most results were as expected: there was a convergence of the *y*-intersects, and the higher the percentage of reactive collision (i.e., lower values of *k*_*m*_) the lower the value of the slope.

From the Lineweaver-Burke plots and, in particular, considering the linear regression model that approximates the equation

(5) $$\begin{array}{c} \frac{1}{V}=\frac{K_{m}}{V_{\max}}\times \frac{1}{\left[\text{S}\right]}+\frac{1}{V_{\max}} \end{array}$$

we were able to estimate the values of *k*_*m*_ and *k*_cat_ described by the simulation parameters (Table 2). Again, it was expected that higher percentages of reactive collision would relate to lower values of *k*_*m*_ and vice versa, whilst the value of *k*_cat_ should be less affected. The presence of negative values for *k*_cat_ and *k*_*m*_ for very low percentages of reactive collisions was unexpected though. This occurs because *k*_cat_ for those situations is supposed to be very close to zero. As this model is stochastic, small variations may lead the values of *k*_cat_ to become negative, hence affecting the values of *k*_*m*_ as well.

To further validate the approximation of the kinetic parameters made by our model we tested them against experimentally validated data. Six enzyme records falling within the range of kinetic values calculated were randomly selected from BRENDA database [41].

**Figure 3:**
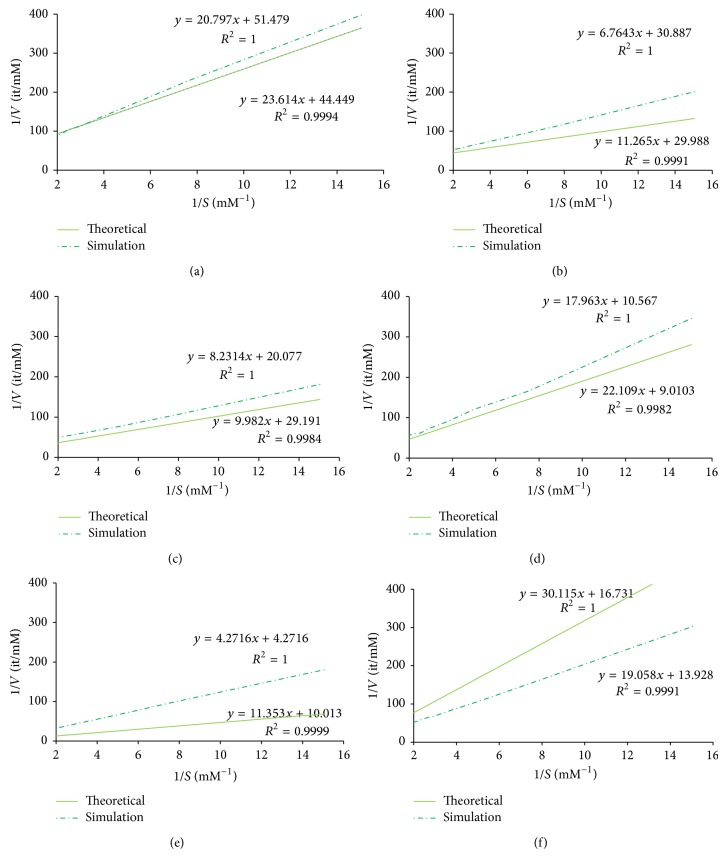
The Lineweaver-Burk plots for experimentally calculated kinetic parameters (represented by a solid line) and simulation parameters simulated by our model (represented by a dash dotted line). From top to bottom, and from left to right, the plots represent the activity of the following enzymes: glutathione reductase, aspartate 1-decarboxylase, alcohol dehydrogenase, IMP dehydrogenase, D-Ala-D-Ala dipeptidase, and pyruvate decarboxylase.

We selected the simulation scenarios producing the most similar approximation to the experimentally validated kinetic parameters (Table 3) and compared the corresponding Lineweaver-Burke plots (Figure 3). As expected, the more similar the approximations were to the real values the better the simulations performed. In particular, the number of time steps stipulated for the duration of the reaction only affects the value of *k*_cat_ and thus can be used to validate the simulation.

### 4.2. A Two-Step Enzymatic Reaction

After validating our model for situations where only one enzymatic reaction occurs, we then studied the behaviour of our simulator when two enzymes are present in a two-stage process.

Specifically, we studied the catalytic activity of two enzymes commonly present in aromatic aldehyde production: the aryl-alcohol dehydrogenase (EC number 1.1.1.90) and the benzaldehyde dehydrogenase (EC number 1.2.1.28).

In particular, the model encompasses the following two equations:

(6) benzyl  alcohol+NAD+⟶benzaldehyde+NADH+H+benzaldehyde+NAD++H2O⟶benzoate+NADH+2H+

The model represented an area of approximately 1 *μ*m^2^, containing a concentration of 1.66 mM (5*E* + 03 molecules) of each type of enzyme, 10^5^ obstacles, and initial concentrations of 3.32*E* + 01 mM and 3.32 mM (1*E* + 05 molecules and 1*E* + 04 molecules) for NAD^+^ and NADH, respectively. During simulation, a concentration of 4.98*E* − 01 mM (1500 molecules) of benzyl alcohol, that is, the substrate of the first reaction, was introduced gradually in the environment, specifically 10% at every 10 time steps.

Agent size and velocity of movement were adjusted according to the molecular weight of the biological species (Table 4). Heavier molecules have a larger radius and a slower diffusion rate. Proportionally, the agents representing benzyl alcohol and benzaldehyde molecules should be two times smaller and faster than the agents representing the molecules of NAD^+^ and NADH.

To facilitate visual inspection, distinct agent types are associated with different colours and proportional sizes. As such, it is possible to visually observe the evolving of the simulation and, at some extent, observe how the agents are moving and interacting with each other. Specifically, it is possible to see how different agents traverse the environment and how behavioural rules are triggered or take precedence over each other.

**Figure 4:**
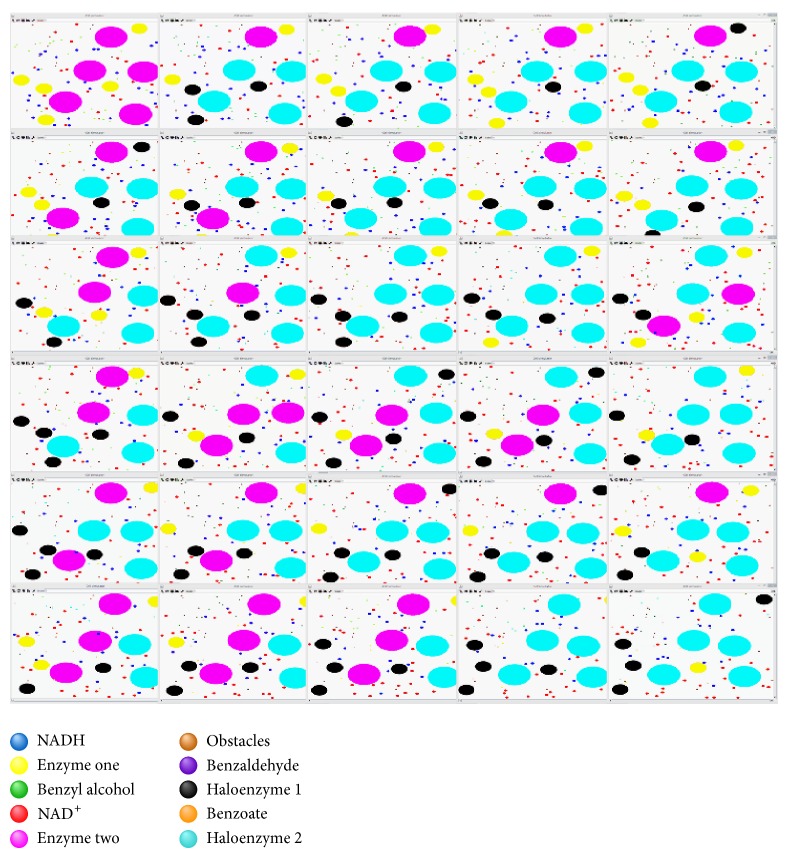
Visual illustration of the evolving of the population of agents during the simulation steps.

As illustrated in Figure 4, although the movement of the molecules of benzyl alcohol (green circles) is quite fast, these molecules are unable to interact with the corresponding enzyme (aryl-alcohol dehydrogenase, named enzyme one and coloured yellow in the figure) unless the enzyme has already bound to a NAD^+^ molecule (i.e., creating the holoenzyme 1, coloured in black). Likewise, interactions with the second enzyme, benzaldehyde dehydrogenase (magenta circles), are limited to the molecules of NAD^+^ (red circles) until the first reaction actually occurs and begins producing benzaldehyde (blue circles). Furthermore, it is possible to observe the reconversion of the molecules of NADH (blue circles) to molecules of NAD^+^ whenever the first hit the cell membrane (i.e., boundary of the environment). Likewise, it is possible to observe that obstacles are playing their part, deflecting the movement of the other agents every so often. A detailed visual documentation of the two-step enzymatic reaction simulation is available in supplementary material (see Supplementary Material available online at http://dx.doi.org/10.1155/2014/769471).

**Figure 5:**
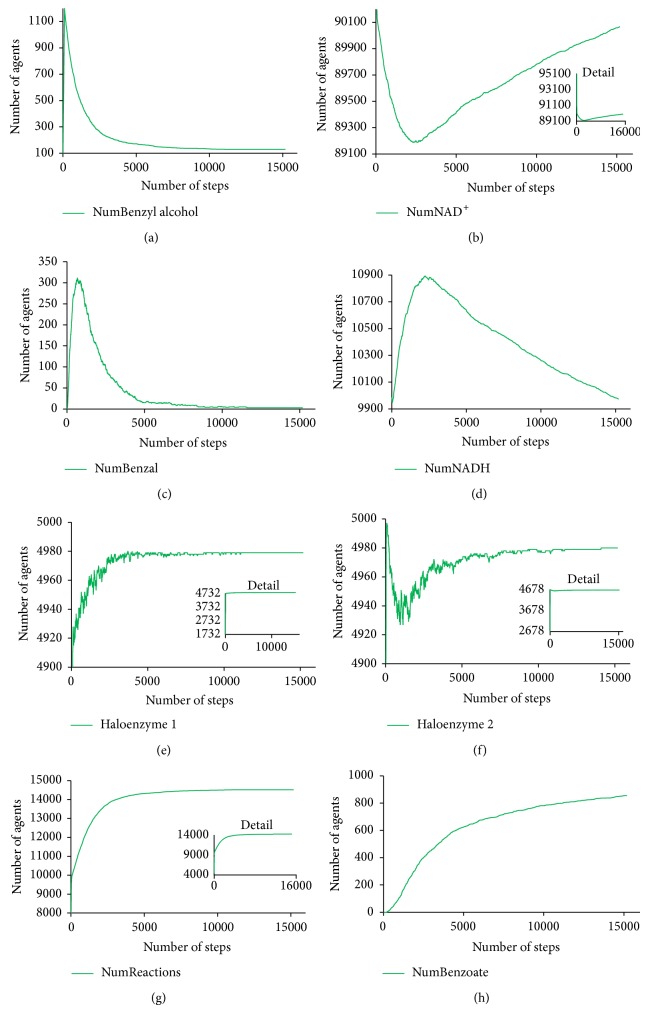
The number of agents of different species interacting in the environment during 15000 simulation steps. From top to bottom, and from left to right, the above plots represent the number of agents of benzyl alcohol, NAD^+^, benzaldehyde, NADH, aryl-alcohol dehydrogenase holoenzyme, and benzaldehyde dehydrogenase holoenzyme. At the bottom, there is the number of occurring reactions and the number of molecules of benzoate excreted the extracellular medium.

Numeric outputs detail these visual insights and present data about the movement of the different species, the velocity at which the reactions are taking place and the behavioural system as a whole (Figure 5). The number of agents representing the first substrate, benzyl alcohol, decays considerably in the first 2000 steps of simulation, together with the disappearance of agents representing NAD^+^ and the creation of agents representing the aryl-alcohol dehydrogenase haloenzyme. This leads to the production of a growing number of agents representing benzaldehyde and an increase in the number of reactions occurring in the system. As soon as benzaldehyde agents start to circulate in the environment, and considering the continuous reconversion of NADH agents into NAD^+^ agents, the second reaction starts to occur. This is reflected in a considerable decrease in the number of agents of benzaldehyde and a fairly proportional increase in the number of the agents representing benzoate, the product that will be ultimately excreted.

### 4.3. System Performance

This work describes the first phase of development of the biomolecular simulator. That is to say that focus was set on identifying and implementing the main biochemical and biophysical laws that would govern the model rather than implementing a realistic picture of the molecular landscape.

As far as we know, there has not been a previous attempt to simulate the effects of spatial localisation and temporal scales of individuals in the modelling of biomolecular systems. So, before engaging into more complex scenarios, it was pivotal to take advantage of available experimental data and ensure that the tool was able to account for basic cellular dynamics, such as those governing enzymes, adequately. Now that we obtained a successful proof of concept, we will work on system scalability in order to address more complex problems.

**Figure 6:**
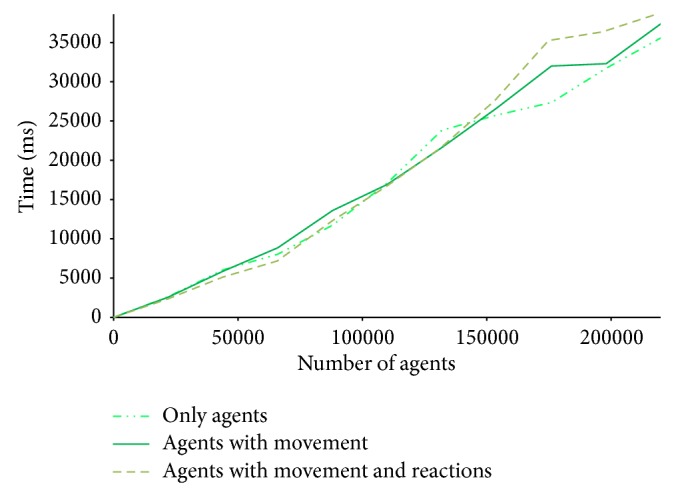
Performance of the tool in scenarios of increasing computational complexity.

For this purpose, we run preliminary performance tests to find out the current scalability of our system.  Figure 6 summarises performance tests using an Intel I7 (2600) 3.4 GHz processor with 6 GB of RAM and running Windows 8 64-bit. The tests accounted for three possible scenarios, as follows: no action, that is, the agents are created but no diffusion or behavioural rules are executed; without reaction, that is, the agents are created and diffusion rules are activated, but agents do not interact among themselves; and, with reaction, that is, agents are created, can move, and can interact. The three scenarios show a similar performance till the system reaches a population of 1.5*E* + 05 agents. That is, till this point, the physics governing agent movement and the introduction of behavioural rules do not affect simulation time significantly. Cost resides on creating the system. After that point, the time taken by the simulation of more elaborated scenarios, that is, more agents in motion and more rules to manage, is somewhat aggravated. Over 2.5*E* + 05 agents, the system becomes computational unviable by a single machine.

So, in the near future, we will investigate the use of distributed and high-performance computing in the simulation of more complex biomolecular systems. Namely, we are investigating the potential of the new distributed environment of MASON, the DMASON (http://www.isislab.it/projects/dmason/), and the Biocellion framework [42].

## 5. Conclusions

ABM is increasingly popular in biology due to its natural ability to represent multiple scales of system decomposition, intertwine complicated behaviours, and deal with spatial-temporal constraints.

The agent-based tool developed in this work aims to support biomolecular simulations and, most notably, provide insights into catalytic efficiency in scenarios of industrial interest. Hence, proof of concept was focused on the approximation of kinetic parameters. The models correctly simulated known enzymatic characteristics and yielded useful predictions that may guide future experimental design. It also provides a simulation variability that may reproduce the experimental variation observed in lab experiments.

Future development of the models presented here will include three-dimensional representation, metabolic pathway simulation, and accounting of extracellular substances. Moreover, we plan to take into advantage the new DMASON platform to engage into distributed, affordable simulation and study more complex scenarios. Other recent high-performance computing frameworks like Biocellion will also be evaluated.

After consolidation, our tool will provide several resources and services for the investigation of bacterial cells in benefit of the research and industry communities.

## Supplementary Material

Supplementary material provides a live demonstration of the biomolecular simulation of the two-step enzymatic reaction. Video “SimulatorDemo” provides general view of the simulator in action while the video “SimulatorDemo_Zoom” provides a closer look into the continuous two-dimensional space, notably over the behaviour of the smaller molecules.

## Figures and Tables

**Table 1:** Agents, behavioural rules, and interacting agents.

Agent	Rules	Interacts with
Enzyme (apoenzyme)	Moving and binding	Metabolite and cofactor
Metabolite	Moving, binding, and death	Enzyme-cofactor complex
Cofactor	Moving, binding, and reconverting	Enzyme and cell membrane
Enzyme-cofactor complex (holoenzyme)	Moving, reaction, and decoupling	Metabolite
Obstacle	Preventing movement	All agents in movement

**Table 2:** Approximation of kinetic parameters by different percentages of reactive collision and time steps.

% reactive collision	Time step	*k*_*m*_ (mM)	*k*_cat_ (s^−1^)
1	0	−0,850576595	−0,028514453
5	0	−2,996153951	−6,87*E* − 01
5	25	−4,656418568	−9,77*E* − 01
5	50	3,439111157	8,08*E* − 01
5	75	2,918555171	6,25*E* − 01
5	100	−21,79373575	−3,92*E* + 00

10	0	−14,6001318	−6,631944616
15	0	11,51508927	7,623330464
25	0	0,87739	1,08*E* + 00
25	5	1,36829	1,44*E* + 00
25	25	2,45375	2,23*E* + 00
25	50	1,20869	1,06*E* + 00
25	75	0,70036	6,22*E* − 01
25	100	0,531257304	4,52*E* − 01

34	1	3,18977	3,93*E* + 00
50	0	0,71725	1,27*E* + 00
75	0	3,65866	6,49*E* + 00
75	5	3,06473	5,33*E* + 00
75	25	1,13387	2,01*E* + 00
75	50	0,60205	1,07*E* + 00
75	75	0,37565	6,69*E* − 01
75	100	0,29693544	5,06*E* − 01

90	0	4,43222	8,17*E* + 00
95	0	4,49601	8,44*E* + 00
95	25	1,277963717	2,396621333
95	50	0,56747	1,11*E* + 00
95	75	0,34196	6,88*E* − 01
95	100	0,237537579	4,88*E* − 01

100	0	0,75547	1,60*E* + 00

**Table 3:** An approximation between experimentally calculated kinetic parameters and the parameters simulated by our model.

Enzyme identification	Experimental kinetics		Simulation parameters		Approximated kinetics	
	*k*_*m*_	*k*_cat_	% reactive collision	Time step	*k*_*m*_ (mM)	*k*_cat_ (s^−1^)
EC 1.8.1.9—glutathione reductase activity	0.404	0.39	25	100	0.531257304	4.52*E* − 01
EC 4.1.1.11—aspartate 1-decarboxylase	0.219	0.65	75	75	0.37565	6.69*E* − 01
EC 1.1.1.1—alcohol dehydrogenase	0.41	1	95	75	0.34196	6.88*E* − 01
EC 1.1.1.205—IMP dehydrogenase	1.7	1.9	25	25	2.45375	2.23*E* + 00
EC 3.4.13.22—D-Ala-D-Ala dipeptidase	1	4.7	75	25	1.13387	2.01*E* + 00
EC 4.1.1.1—pyruvate decarboxylase	1.8	1.2	25	5	1.36829	1.44*E* + 00

**Table 4:** The weight, size, and diffusion rate of the molecules represented in the two-step enzymatic system.

Species	Molecular weight (g/mol)	Particle radius (*µ*m)	Diffusion rate (*µ*m^2^/s)
Benzyl alcohol	108.14	0.323 × 10^−3^	4.018 × 10^−14^
NAD^+^	661.41	0.657 × 10^−3^	1.975 × 10^−14^
NADH	663.43	0.658 × 10^−3^	1.973 × 10^−14^
Benzaldehyde	106.121	0.321 × 10^−3^	4.047 × 10^−14^
Benzoate	121.12	0.338 × 10^−3^	3.843 × 10^−14^
